# Results of Ranibizumab Treatment of the Myopic Choroidal Neovascular Membrane according to the Axial Length of the Eye

**DOI:** 10.1155/2020/3076596

**Published:** 2020-05-12

**Authors:** Alexandr Stepanov, Martin Pencak, Jan Nemcansky, Veronika Matuskova, Marketa Stredova, David Beran, Jan Studnicka

**Affiliations:** ^1^Department of Ophthalmology, Faculty of Medicine in Hradec Kralove, Charles University in Prague and University Hospital Hradec Kralove, Hradec Kralove, Czech Republic; ^2^Department of Ophthalmology, Third Faculty of Medicine, Charles University in Prague and University Hospital Kralovske Vinohrady, Prague, Czech Republic; ^3^Department of Ophthalmology, Faculty of Medicine, University of Ostrava and University Hospital Ostrava, Ostrava, Czech Republic; ^4^Department of Ophthalmology, Faculty of Medicine, Masaryk University Hospital, Brno, Czech Republic

## Abstract

**Aim:**

A retrospective evaluation of the results of treatment of myopic choroidal neovascularization (mCNV) with intravitreal injections of ranibizumab in a *pro re nata* (PRN) regimen in three groups of patients distributed according to axial length.

**Methods:**

The paper presents a retrospective multicenter study carried out with the cooperation of several Departments of Ophthalmology in the Czech Republic. The study included 60 eyes of 60 patients suffering from mCNV, divided according to axial length into three groups. The first group consisted of 20 patients with an axial length of the eyes shorter than 28 mm (Group 1), the second group included 27 patients with axial lengths ranging from 28 mm to 29.81 mm (Group 2), and 13 patients had axial lengths longer than 30 mm (Group 3). All patients were first administered 3 initial intravitreal ranibizumab injections at monthly intervals (loading phase), and other injections were administered according to a PRN treatment regimen. Patients were evaluated before treatment and then at intervals of 3, 6, 9, and 12 months. The effect of ranibizumab treatment on the functional and morphological parameters of the affected eye was evaluated.

**Results:**

The average baseline BCVA ± SD in Group 1 was 52.6 ± 12.5 letters of ETDRS optotypes, and at the end of the one-year follow-up, it was 63.3 ± 11.8 letters. The average baseline of CRT ± SD in this group was 377.4 ± 80.0 *μ*m, and in the 12th month, it was 311.1 ± 63.7 *μ*m. The average baseline BCVA ± SD in Group 2 was 50.2 ± 9.0 ETDRS letters, and at the end of the follow-up, it was 60 ± 12.4 letters. The average baseline of CRT ± SD in Group 2 was 391.2 ± 85.2 *μ*m, and in the 12th month, it was 323.9 ± 91.2 *μ*m. In Group 3, the average baseline of BCVA was 48.5 ± 14.5 ETDRS letters, and at the end of the one-year follow-up, it was 55.7 ± 16.1 letters. The average baseline CRT ± SD for Group 3 was 342.1 ± 94.9 *μ*m, and after 12 months, it was 287.8 ± 88.4 *μ*m. An improvement of BCVA by ≥15 letters of ETDRS optotypes was achieved by 3 patients of 20 (15%) in Group 1, by 5 patients of 27 (18.5%) in Group 2, and by 3 patients of 13 (23.1%) in Group 3. All these changes were statistically significant in comparison with the input values (*p* < 0.05).

**Conclusion:**

Ranibizumab treatment in patients with mCNV in our study resulted in statistically significant improvement in BCVA and a decrease in CRT in all groups of patients. Our results from a routine clinical practice correspond with the results of large clinical studies; we confirm a particularly good effect of treatment in patients with axial lengths of the eye smaller than 28 mm.

## 1. Introduction

Myopia is the most frequent cause of decreased visual acuity in the total population, particularly in East Asia, where it affects approximately 40% of adults aged over 40 years [1]. Pathological myopia is the most serious form of myopia, and its definition includes a refractive error of minimally −6.0 dioptres or axial length of the bulbus of 26 mm and more, accompanied by degenerative changes in the sclera, choroid, and retina [2–4].

Choroidal neovascularization based on pathological myopia (myopic CNV) is one of the most serious complications of pathological myopia in patients of the productive age, with a prevalence of 0.04% to 0.05% in the total population [5, 6]. It develops as a result of the mechanism of wound healing following ruptures of Bruch's membrane and represents the most dangerous sight-threatening event in pathological myopia.

The prevalence of myopic CNV is estimated to be 0.05% among patients older than 49 years in the Blue Mountains Eye Study [7] and 0.04% in patients over 40 in the Peking ophthalmic study [8]. The prevalence also differs according to population and demographic characteristics. According to data from the United States, 5.2% patients with axial lengths higher than 26.5 mm showed the signs of myopic CNV [9], whereas in Japan manifestations of mCNV were recorded in 11.3% eyes with refraction higher than −8 D or axial lengths more than 26.5 mm [10]. It has been reported that myopic CNV occurs more frequently in women, and the prevalence in the female population ranging between 52% and 87.7% [11].

Ranibizumab is a recombinant humanized monoclonal antibody of size 48 kDa lacking the Fc fragment [12]. The safety and effectiveness of intravitreal treatment with ranibizumab in the case of myopic CNV has been demonstrated in several clinical studies [13–19]. Ranibizumab is the first anti-VEGF preparation approved in many countries throughout the world for the treatment of visual affection due to myopic CNV, and it is recommended as the first-choice drug [20].

This study evaluates the treatment of intravitreally administered ranibizumab in a *pro re nata* (PRN) regimen in patients with myopic CNV, distributed according to axial lengths into three subgroups: less than 28 mm, 28-29.9 mm, and more than 30 mm.

## 2. Methods

### 2.1. Selection of Patients

**Figure 1:**
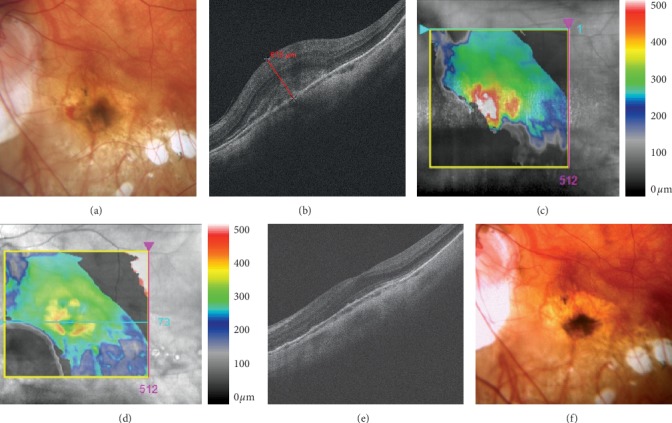
Choroidal neovascularization in a patient with pathological myopia. (a) Fundus photography before Lucentis treatment. Acute neovascular membrane and subretinal hemorrhage in the nasal part. (b-c) HD-OCT before treatment. Hyperreflective tissue grows through retinal pigment epithelium. Central retinal thickness is 675 *μ*m. (d-e) HD-OCT after treatment. A decrease of edema and improvement of foveolar depression. (f) Fundus photography after treatment. Reduction of neovascular membrane, and hemorrhages are not presented.

This was a multicenter retrospective observational study from a routine clinical practice in the University Hospital in Hradec Kralove, Masaryk University Hospital in Brno, University Hospital in Ostrava, and University Hospital in Kralovske Vinohrady, Prague, which took place in the period from July 2017 to August 2019. The criteria for inclusion were as follows:

Pathologic myopia with an axial length of 26 mm or more, presence of active subfoveal or juxtafoveal CNV, which was demonstrated by means of fluorescent angiography (FA), and a one-year follow-up period (Figure 1). The criteria for exclusion were as follows: CNV resulting from causes other than myopia (e.g., age-related macular degeneration (AMD), central serous chorioretinopathy, diabetic retinopathy, retinal vein occlusion, vasculitis, and uveitis), previous treatment of CNV (including photodynamic therapy (PDT) and intravitreal injections of anti-VEFG drugs), and other possible causes of decreased BCVA (e.g., advanced cataract and other disease of the retina and/or the anterior segment). All included patients were treatment naïve, and no one patient was bilaterally affected. The baseline BCVA ranged between 75 and 25 letters of the ETDRS optotypes (Snellen 20/32–20/320) on the affected eye.

### 2.2. Data Collection

In the course of the one-year follow-up, BCVA was measured in all patients, and slit-lamp examination, biomicroscopy in artificial mydriasis after instillation of 0.5% tropicamide, and optical coherence tomography (OCT) by means of an OCT Cirrus 4000 (ZEISS, Oberkochen, Germany) were performed. These examinations were carried out at each ward round: prior to the commencement of treatment and in the 3rd, 6th, 9th, and 12th month. The axial length of the eye was measured only at the first visit by means of a Zeiss IOL Master 500 Biometry A Scan apparatus or a NIDEK US-4000 Ultrasound apparatus. FA was performed at the first visit (Visucam 500, ZEISS, Oberkochen, Germany) and in cases of doubts in the assessment of disease activity. BCVA was determined by means of standardized ETDRS optotypes in all centers. CRT was defined as the distance between the internal limiting membrane and RPE in the fovea. All patients signed the form of informed consent prior to administration of the intravitreal injections. The protocol of the study observed the principles of the Helsinki Declaration. We did not evaluate fundus differences among eyes in the whole group of patients (diffuse atrophy, tessellate fundus, patchy atrophy, lacquer cracks, etc) despite the fact that it is an interesting consideration. Surgical intervention was performed in an operating room. Preparation of the drug and its administration took place under aseptic conditions. Ranibizumab (0.5 mg in 0.05 ml) was administered under local anesthesia using a 30-gauge needle trans-sclerally 3.5 mm (in pseudophakic eyes) to 4.0 mm (in phakic eyes) from the limbus.

### 2.3. Criteria for Readministration

Ranibizumab treatment was applied in the PRN dosing regimen, i.e., the first three injections were followed by controls with possible addition of another injection if the signs of CNV activity continued according to the condition on OCT (intra- and subretinal fluid and RPE ablation) and also if new macular hemorrhage was observed. FA was performed in cases of doubts in the assessment of disease activity, when the signs of CNV activity have been demonstrated on the basis of increasing hyperfluorescence (leakage).

We have chosen the loading dose consisting of three-month intravitreal injections to get the greatest effect both anatomically and functionally because we knew that the follow-up controls would follow every three months, which was the most achievable interval in real clinical practice. The system of quarterly follow-up visits is based on the possibilities in real clinical practice and patient's ability to undergo these controls. Evaluated were the changes in BCVA on ETDRS optotypes, CRT development according to OCT, and the safety profile of the preparation. Although this was a retrospective multicenter study, the retreatment criteria were the same everywhere and resulted from the treatment conditions in real clinical practice at all centers.

### 2.4. Statistical Analysis

Statistical analysis was performed by means of the software IBM SPSS Statistics 23. Quantitative data are expressed by the mean and extent. BCVA and CRT values were analysed using the Wilcoxon test. Changes in BCVA and CRT were evaluated by means of the paired *t*-test. Pearson's correlation coefficient was employed to evaluate the correlation between the axial length and the resultant BCVA and CRT. Statistical significance was defined as *p* < 0.05.

## 3. Results

The study included altogether 60 eyes in 60 naive patients who were examined in four Departments of Ophthalmology in the Czech Republic. The baseline demographic data of patients are presented in Table 1. All patients were treated with ranibizumab. The patients were divided into 3 groups according to the axial length. Group 1 consisted of 20 eyes (33.3%), Group 2 consisted of 27 eyes (45%), and Group 3 included 13 eyes (21.7%). The average axial length of the eyes in Group 1 was 27.08 ± 0.6 mm (range 26–27.9 mm), in Group 2 28.83 ± 0.55 mm (range 28–29.81 mm), and in Group 3, 31.61 ± 1.74 mm (range 30–35.1 mm). In Group 1, the average age of patients was 62 years (range 36–84 years), of whom 15 were women (75%), the average age of Group 2 was 63.1 years (range 35–83), of whom 25 were women (92.6%), and in Group 3, the average age was 55.5 years (range 28–80) and included 8 women (61.5%). The average number of intravitreal injections of ranibizumab was 4.0 ± 1.5 in the whole cohort: 3.88 ± 1.3 in Group 1, 3.59 ± 1.6 in Group 2, and 2.9 ± 1.3 in Group 3 (*p*=0.14). No subsequent complications related to intravitreal ranibizumab administration were observed, e.g., serious intraocular inflammation, hemophthalmus, or development of secondary glaucoma.

### 3.1. Analysis of Visual Acuity

**Figure 2:**
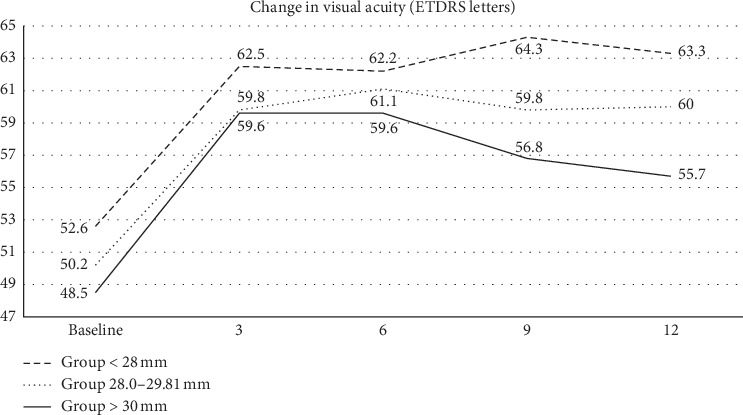
Functional outcomes over time for eyes with mCNV with different axial lengths. All groups showed BCVA improvement from baseline to the final 12-month follow-up; however, the group with axial length <28 mm showed the greatest letter gain.

The baseline BCVA ± SD in the total cohort of patients was 51.0 ± 11.5 letters of ETDRS optotypes: in Group 1, it was 52.6 ± 12.5 letters, 50.2 ± 9.0 letters in Group 2, and 48.5 ± 14.5 letters in Group 3 (*p*=0.182). The development of BCVA is represented in Figure 2. The final BCVA in all patients at the end of the follow-up was 60.0 ± 13.2 letters of ETDRS optotypes: 63.3 ± 11.8 letters in Group 1, 60 ± 12.4 in Group 2, and 55.7 ± 16.1 in Group 3 (*p*=0.14).

**Figure 3:**
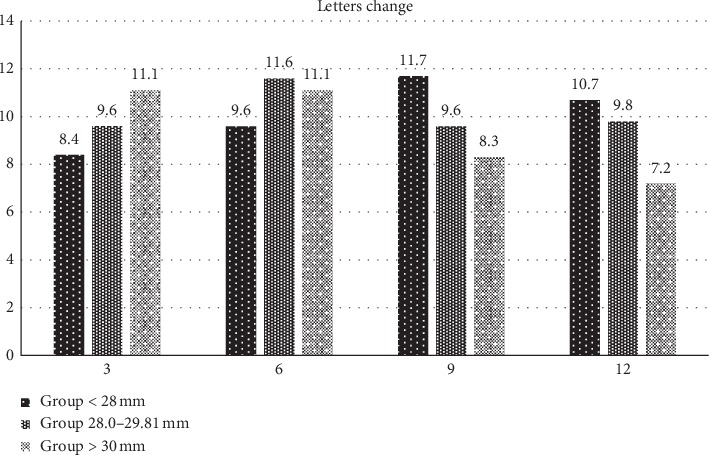
The mean change in ETDRS letters from baseline for three groups with different axial lengths during the first year of ranibizumab treatment.

The changes relative to the baseline value were statistically significant in the course of the whole follow-up (*p* < 0.05) (Figure 3). Improvements in BCVA by ≥15 letters of ETDRS optotypes were achieved by 3 patients of 20 (15%) in Group 1, by 5 patients of 27 (18.5%) in Group 2, and by 3 patients of 13 (23.1%) in Group 3.

A decrease in BCVA by ≥ 15 letters of ETDRS optotypes was found in four patients during the first 6 months of the follow-up as the result of the following changes: one case of RPE atrophy in the macula, one case of development of a macular hole, and one case where the patient underwent a laser refractive surgery with gradual development of haze. An RPE rupture developed in one patient immediately after the first injection of ranibizumab. These patients were excluded from further evaluation.

A comparison of the results of changes in BCVA after one-year follow-up has not revealed a statistically significant difference between groups. The difference between the first and second group was 3.3 letters of ETDRS optotypes (*p*=0.22), between the second and third, 4.3 letters of ETDRS optotypes (*p*=0.19), and between the first and third, 7.6 letters of ETDRS optotypes (*p*=0.11).

### 3.2. Anatomical Results

**Figure 4:**
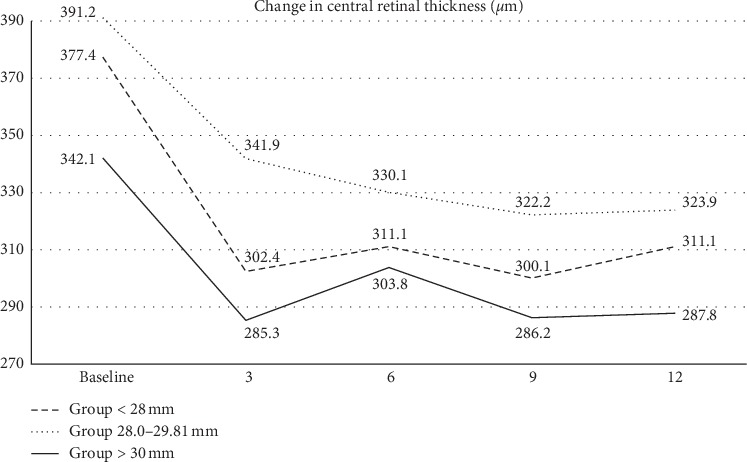
Anatomical outcomes over time for eyes with mCNV with different axial lengths. All groups showed a significant decrease in CRT from baseline to the final 12-month follow-up.

**Figure 5:**
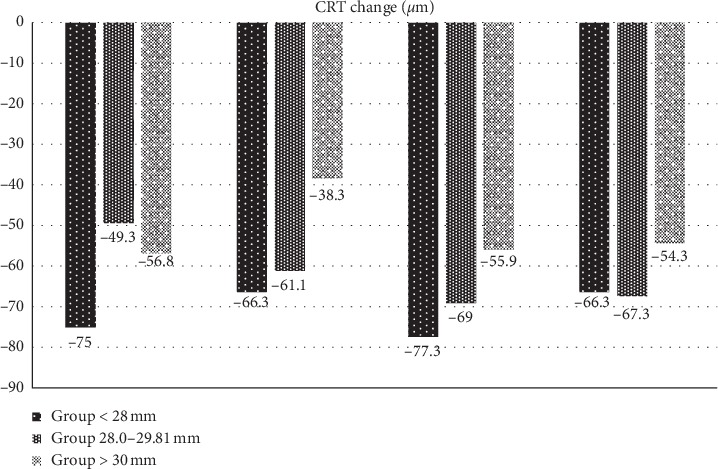
The mean change in CRT in micrometres from baseline for three groups with different axial lengths during the first year of ranibizumab treatment.

The baseline value of CRT ± SD in the total cohort of patients was 376 ± 86.2 *μ*m: in Group 1, 377.4 ± 80 *μ*m, in Group 2, 391.2 ± 85.2 *μ*m, and in Group 3, 342.1 ± 94.9 *μ*m (*p* = 0.3). The development of CRT values is represented in Figure 4. At the end of the follow-up in the 12th month, CRT ± SD in the total cohort was 312 ± 82.9 *μ*m: in Group 1, 311.1 ± 63.7 *μ*m, in Group 2, 323.9 ± 91.2 *μ*m, and in Group 3, 287.8 ± 88.4 *μ*m (*p*=0.176) (Figure 5). At the end of the follow-up, residual macular edema (intraretinal and/or subretinal) was found in 16.7% of all patients, of whom 4 patients were from Group 1 (6.7%), 5 from Group 2 (8.3%), and 1 from Group 3 (1.7%).

### 3.3. Correlation between Axial Length and Final BCVA and CRT

After a one-year follow-up period, the correlation between the axial length and the final average value of BCVA and CRT was evaluated using Pearson's correlation coefficient. For the total cohort, this value for correlation of axial length and BCVA was −0.19 and for axial length and CRT was 0.16. This means that the higher axial length of the eye related to the smaller final gain of BCVA and the smaller decrease in CRT.

## 4. Discussion

A number of clinical studies have described the benefit of anti-VEGF treatment in patients with mCNV [13–22]. These papers have demonstrated that in the course of a one-year follow-up after treatment with ranibizumab, 65–92.7% eyes with mCNV resulted in an improvement in BCVA of at least 5 letters of ETDRS optotypes, which is better than in the case of treatment using PDT. The present retrospective study has evaluated the functional and anatomical results of ranibizumab treatment, in the PRN regimen, of naive eyes with mCNV distributed into 3 subgroups according to axial lengths. The cohort consisted of a relatively large group (*n* = 60) of patients, and BCVA improvement in the total cohort at the end of a one-year follow-up was +9.0 letters of ETDRS optotypes; on average, 4.0 ± 1.5 injections were administered.

The randomized, multicenter, double-blind study Radiance evaluated two individualized application regimens of ranibizumab treatment in 277 patients with mCNV (in the first group, patients received two introductory injections followed by treatment in the PRN regimen; in the second group only one introductory injection was administered) [19].

On the basis of the results of the study, it is evident that no statistically significant differences have been demonstrated in the efficiency of treatment between the first and second groups at the end of a one-year follow-up. The average improvement in BCVA was +13.8 letters of ETDRS optotypes in Group 1 (the average number of ranibizumab injections being 4.0), and in Group 2, the average improvement was +14.4 letters (the average number of ranibizumab injections being 2.0). At the final checkup in the 12th month, 64.2% of all patients showed no signs of CNV activity.

The present study has recorded a gain in the total cohort on average by 9.0 letters of ETDRS optotypes after 12 months from the beginning of treatment, with a median of 3.0 injections in the course of a one-year follow-up. Our results could be influenced by a smaller number of patients and worse baseline values of BCVA (51.0 letters of ETDRS optotypes in the total cohort versus 55.8 letters in the Radiance study).

The Radiance study is for the time being the only study evaluating the results of ranibizumab treatment according to the axial length of the eye in myopic patients during a one-year follow-up [23]. In the first group with axial lengths <28 mm (41 patients), an average gain of +16.8 ETDRS letters was recorded, with the average number of injections 3.95. In the second group with axial lengths of 28–30 mm (34 patients), the average gain was +13.6 ETDRS letters (2.8 injections). The third group consisted of individuals with axial length more than 30 mm (30 patients), in which the average gain was +13.4 ETDRS letters with an average number of 3.8 injections in a year.

In agreement with the Radiance study, we have found that the largest gain in BCVA was in the first group of patients (axial length <28 mm) and the smallest gain was in the third group (axial length >30 mm). This finding may be related to the prevalence of degenerative changes in the macula, which are more represented in the eyes with a larger axial length, and which do not make possible such improvement as was observed in the group with a smaller axial length.

Another possible explanation is the assumption that the larger eyes need a higher dose of intravitreal drug administration.

The present study has demonstrated in the first group of patients a gain of 10.7 ETDRS letters after 12 months from the commencement of treatment with an average number of 3.88 injections; in the second group, the gain was 9.8 ETDRS letters (average number of injections 3.59), and in the third group, the gain was 7.2 ETDRS letters (average number of injections 2.9). Comparison of the results of BCVA changes after a one-year follow-up and did not reveal a statistically significant difference between the groups, but indirect dependence was demonstrated between axial length and the resultant value of visual acuity.

The results of the study also correlate with the results of the paper by Wu and Kung [18], who published results of a one-year follow-up of patients with mCNV treated with ranibizumab in the PRN regimen. The average axial length of the eye was 28.24 ± 1.09 mm (in the range 26.07–29.63), and the average number of ranibizumab injections was 3.44 ± 0.92 (in the range 3–6). During the 12 months, in 19 eyes, only 3 introductory ranibizumab doses were administered (76%). The other 6 eyes (24%) needed between one and three more ranibizumab injections in the course of the follow-up. After a 12-month follow-up, an average improvement in BCVA of +14.4 letters of ETDRS optotypes (*p* < 0.001) with a decrease in CRT of −47.6 mm (*p*=0.012) was observed. The baseline values of axial length, average number of ranibizumab injections, and functional and anatomical results in our study are comparable with the study by Wu et al., and they have demonstrated a clinically significant improvement of BCVA and a decrease in CRT during a one-year follow-up.

Clinical trials are by their very nature carried out on a restricted study population. Despite this, the results of such trials are widely assumed to reflect outcomes that may be hoped to be achieved in future clinical practice.

The strengths of our study include evaluating the effect of ranibizumab treatment in groups of patients divided by axial lengths. Another strong point of our study is the multicenter design. Patients were enrolled in four university municipal hospitals throughout the Czech Republic. The limitations of our study are the retrospective and observational nature and the relatively small sample size compared with bigger clinical trials.

## 5. Conclusion

The study presents one-year real-life outcomes in treatment-naive patients with myopic CNV divided into three groups according to axial length of the eye and treated with ranibizumab in a PRN regimen. According to our experience, ranibizumab treatment with a *pro re nata* regimen results in a statistically significant visual acuity gain and improvement in retinal anatomic outcomes over a one-year follow-up in all groups of patients. The group with axial lengths >30 mm demonstrated a poorer functional and anatomical response to the treatment.

When comparing the results of BCVA and CRT changes after one year of follow-up, there was no statistically significant difference between groups. We have demonstrated that there exists an indirect dependence between the axial length of the eye and the resultant BCVA and also an indirect dependence with a decrease in CRT in the course of one-year treatment.

## Figures and Tables

**Table 1:** Baseline demographic, anatomical, and vision characteristics of all groups of eyes separated by axial length.

	All patients 26.0–35.1 mm (*n* = 60) (100%)	Group 1 <28 mm (*n* = 20) (33.3%)	Group 2 28.0–29.81 mm (*n* = 27) (45%)	Group 3 >30 mm (*n* = 13) (21.7%)	*p*
Mean age (years)	61 ± 14.3	62 ± 15	63.1 ± 12.5	55.5 ± 16.3	0.240
Female, no.	15 (75%)	15 (75%)	25 (92.6%)	8 (61.5%)	0.431
CRT (*μ*m)	376 ± 86.2	377.4 ± 80	391.2 ± 85.2	342.1 ± 94.9	0.325
BCVA (ETDRS)	51.0 ± 11.5	52.6 ± 12.5	50.2 ± 9	48.5 ± 14.5	0.182

## Data Availability

The data used to support the findings of this study are freely available and are included within the article.
